# Supplementary material for: Are carbon emissions trading and green financial instruments synergistic? -Comprehensive quantitative research based on content analysis
Source: PLoS One. 2024 Mar 7;19(3):e0298601. doi: 10.1371/journal.pone.0298601 (PMC10919601; doi:10.1371/journal.pone.0298601)
Supplement: S1 Data — (ZIP) [file pone.0298601.s001.zip › Data/地级市Z_F工作报告.zip!/╕ú╜¿/╧├├┼╩╨/└┤╫╘╣1⁄2╓┌║┼í╛┬φ┐╦╩2╛▌═°í┐_2010.docx]

各位代表：

　　现在，我代表市人民政府，向大会作政府工作报告，请予审议，并请市政协委员和各位列席人员提出意见。

　　一、2009年工作回顾

　　2009年是新中国成立60周年，也是我市发展历史上很不平凡的一年。一方面，百年不遇的金融危机给我市发展带来了严重冲击和挑战；另一方面，海西战略上升为国家战略和两岸关系和平发展给我们带来了重大历史机遇。在市委的正确领导下，我们坚持以邓小平理论和“三个代表”重要思想为指导，深入学习实践科学发展观，应对危机全力落实“三保”，抓住机遇努力“先行先试”，全市上下凝心聚力、坚定信心，奋发有为、开拓创新，经济回升向好趋势不断巩固，质量效益进一步提升；对台交流合作蓬勃开展，前沿平台作用进一步显现；重点建设、岛外开发、政策争取、城市管理等工作积极有效，中心城市的综合优势进一步增强；社会事业、民生保障、生态建设、社会管理和公共服务继续加强，社会和谐安宁。初步统计，全年地区生产总值1619亿元，增长8%（按可比价格计算）；财政总收入451.4亿元，其中地方级财政收入240.5亿元，分别增长10.1%和9.2%，全社会固定资产投资882亿元，三项指标均提前一年完成“十一五”目标；城镇居民人均可支配收入26131元，增长9.1%；农民人均纯收入9153元，增长8%；居民消费价格指数97.3%，城镇登记失业率4.01%，人口自然增长率7.85‰。单位生产总值能耗降低2.5%以上，节能减排完成年度目标。一年来，我们主要做了以下工作：

　　（一）积极应对金融危机，企稳回升势头更加向好

　　我市经济外向度高。受国际金融危机影响，我市企业自2008年9月份以来普遍遇到了市场萎缩、订单减少、成本增加、利润缩减、风险加大等一系列困难，全市经济增速大幅下滑。面对严峻形势，一是及时做出了“保增长，工业首当其冲；保增长，三产大有可为；保增长，扩大投资拉动；保增长，大力招商引资；保增长，高度重视外贸；保增长，帮扶服务企业”的部署。同时，认真贯彻上级“保增长”的一系列政策，结合我市实际出台64个政策性文件，在企业融资、开拓市场、扶持中小企业、进出口贸易、促进房地产健康发展等方面提供支持。二是组织开展以“帮助企业树立信心、帮助企业开拓市场、帮助企业解决信贷资金、帮助企业调整结构、帮助企业加快技改”等“五帮”为主要内容的“走进和服务千家企业”活动。全年完成出口退税180亿元，增长18%；市财政直接扶持出口资金1.85亿元；增值税转型以及各种税费减免减轻企业负担18.7亿元；全市金融贷款余额2989.6亿元，增长26.2%，其中中小企业贷款增长22.5%。出口信用保险承保额突破30亿美元，增长1.3倍，对我市一般贸易出口覆盖率达到19.6%；出口企业获得信保赔款和受助追回欠款近3000万美元；获得保单项下融资6.8亿美元，增长129%。开展各种购物节、展销、政府采购、“家电、汽车、摩托车下乡”等促销活动196场次。三是积极向上争取政策。有89个项目列入国家增投计划；争取国家外贸扶持资金1.5亿元。同时积极帮助企业向上争取产品退税和提高退税率。上述措施有效改善了企业生产经营环境，扭转了经济下行的局面，促进了全市经济较早实现企稳回升。地区生产总值增速从一月份－6.9%到全年的8%，回升14.9个百分点；外贸进出口指标持续好于全国、全省水平，对全省的贡献加大，进出口总量占全省的比重从去年的53.5%提高到54.4%；规模以上工业在产值增速持续回升的同时，运行的质量效益一直较好，经济效益综合指数保持高位，全年达175，同比提高5个百分点。

　　（二）大力推进产业发展，持续增长基础更加扎实

　　坚持把保增长与调结构相结合，大力实施项目带动战略，一大批有规模、高质量的产业项目加快实施，产业结构进一步优化。三次产业比例1.3∶48.5∶50.2，第三产业增加值增长14.5%，占三次产业比重提高4个百分点。一是加强重点产业发展规划。大力发展先进制造业和现代服务业，把培育平板显示、计算机与通讯设备、输配电及控制设备、生物与新医药等13条产值百亿至千亿的产业链（群）作为先进制造业发展的重点，把总部经济、港口物流、文化创意等10个行业作为现代服务业的发展重点，已经编制发展规划并组织实施。二是扎实抓好一批龙头骨干项目。冠捷科技、景智光电、许继高压、ABB增资、松下电子增资、金桥卷烟技改、厦顺铝箔技改扩、LGD和冠捷科技合资、戴尔手机等项目进展顺利，全市完成工业投资165.2亿元，净增规模以上工业企业212家。国家太阳镜生产基地落户厦门。宸鸿科技、麦克奥迪等一批企业逆势增长，客车、助听器、电器开关等国际市场进一步拓展。服务业方面，引进了嘉里物流、联邦快递、柯达物流中心、中国医药集团物流中心、吉祥航空、香港快运航空等企业和项目，厦门机场始发和经停航线新增20条，厦门口岸出入境人员突破300万人次；五缘湾、观音山、新站等商务营运中心区引进一批企业的地区总部、销售中心、结算中心和研发中心；厦门银行、君龙人寿、厦门证券、国贸期货等企业加快异地拓展，东莞证券、国信期货等在厦新设营业部，5家企业在境内外上市，2家企业获批即将上市；新增软件企业108家，软件业产值达230亿元；温德姆、佰翔等高星级酒店开业，“闽南神韵”旅游一台戏实现公演，五缘湾游艇帆船基地、双龙潭体育公园等一批项目顺利推进，全市接待游客突破2500万人次，增长15.1%；全年举办展会1700多场次，荣膺“全国十大会议目的地”，“石材展”成为世界第二大石材专业展。三是加强创新体系建设，服务产业发展。成立厦门产业技术研究院、食品检测中心和食品科技孵化园，国家级LED检测中心基本建成，中科院城市环境研究所通过验收。新认定高新技术企业303家，新增2个国家级、22个省部级科研机构，联想移动公司获国家级企业技术中心认证，宏发电器、雅迅网络成为国家创新型企业。实施标准化战略，参与制定9个国家标准项目、7个行业标准项目。“创建知识产权示范城市”通过评估，全市发明专利授权量增长43.8%。启动首届厦门市政府质量奖，新增9个中国驰名商标。实施教育服务产业发展行动计划，厦门理工学院成为全省惟一的国家级创新工程师培养与实训基地，火炬高新区成为全省惟一的国家级海外高层次人才创新创业基地。

　　（三）发挥前沿平台优势，两岸交流合作更加密切

　　抓住两岸关系和平发展的历史机遇，落实国务院支持海西建设的《若干意见》，充分利用各种有利条件，发挥前沿平台优势，积极推进、广泛开展对台交流合作。一是人员往来更加便利。口岸软、硬件建设加强，完成厦金客滚泊位改造工程，率先开辟两岸客货滚装运输新航线并实现班轮化运作。厦金航线试行台湾至厦门行李直挂通关托运。厦航在台湾设立分公司。厦门航点至台湾空中直航每周班次达16个，并实现腹舱载货。厦金“小三通”每日航班增至32个，全年进出旅客达120万人次。实现厦金邮件总包直封、厦门－台北邮件直封直达。在全国率先启动台湾居民来往大陆签注自助受理业务。二是产业对接更有成效。确定了先进制造业、软件与信息服务、金融保险、服务外包、旅游会展、航运物流、文化创意、商贸、邮政通信、农业种苗与农产品加工等十个重点对接合作领域，规划、实施大陆中药材输台物流口岸等一批对接项目。成为全国首个国家级对台科技合作与交流基地，成立了全国首个海峡两岸农产品检验检疫技术中心。全年实际利用台资（含第三地）6.42亿美元，对台进出口总额42亿美元。富邦证券在厦设立代表处，富邦财险获批筹建。大陆首家台资旅行社——厦门灿星国际旅行社开业。大嶝对台小额商品交易市场改扩建、厦门闽台农业高新技术园区、台湾种苗引繁示范基地建设顺利。厦门口岸进口的台湾水果占全国总量七成，进口量和货值超过前四年总和。三是交流活动更加广泛。成功举办首届海峡论坛，成为继国共论坛、“两会”协商之后以基层、民众交流为特点的第三个两岸交流平台；成功举办首次厦金海峡横渡、海峡杯帆船赛等一系列体育赛事，成为全国第一个对台体育合作与交流基地；厦门市政府代表团成功参访台湾，成为大陆第一个以地方政府名义组团和第一个由政府市长率团的赴台参访团。同时，在全国率先开展在厦台湾地区居民专业技术职务任职资格评审试点。台交会、文博会、图书交易会、保生慈济文化节、郑成功文化节、农渔业论坛、民间艺术节、中医药合作交流研讨会等活动顺利圆满举行。

　　（四）加快城市建设，发展环境更加完善

　　按照建设现代化国际性港口风景旅游城市和海峡西岸重要中心城市的定位，加快拓展城市发展空间，加强重大基础设施建设，落实高起点、高标准、高层次、高水平要求，与中心城市相匹配的承载、辐射、带动功能进一步增强。一是建设大港口、大交通。福厦高速铁路建成通车，厦深、龙厦铁路建设顺利推进，厦门新站主站房主体完工，老站房改造方案基本确定，厦门迎来高铁时代。沈海高速扩建、厦门至南安（金淘）高速、厦成高速以及岛内、岛外、连接岛内外的重要路网项目加快建设，我国第一条深海隧道——翔安隧道全线贯通；成功大道、翔安大道二期、丙洲大桥等建成通车；环岛干道、海翔大道、海沧疏港通道以及BRT一、二号线延伸段等项目按计划推进；海沧大桥西引道等项目开工。东渡现代码头、海澳石化等5个泊位、海沧航道扩建二期、“海上一日游”航道、五通至金门水头航道等项目顺利实施。以港口为核心的现代化立体式综合交通枢纽初步形成，厦门港货物吞吐量突破一亿吨，成为海西首个亿吨大港；厦门机场旅客吞吐量突破一千万人次；厦航成为国内第六家年客运量超千万的航空公司。二是加快重大片区开发和产业园区建设。环东海域、五缘湾、杏林湾、湖边水库、厦门新站等重点片区开发顺利推进，规划总面积167.6平方公里，累计完成投资400亿元；火炬（翔安）高新区、集美机械工业集中区、同安工业集中区、环东海域产业园等基础设施和配套建设进一步完善。启动了新城规划，开展了厦门新机场、第二东西通道、城市轨道交通等重大基础设施项目前期工作。三是加强市政基础设施。翔安隧道供水管道、220千伏金榜输变电、电力进岛第一通道扩建、东部燃气电厂、天然气利用及天然气转换、石胄头等污水处理厂改扩建、天地湖和筼筜湖污水截流等一批项目竣工或基本建成。梧村汽车站以及一批停车场和公交场站投入使用。突出安全饮用水工程建设，汀溪水库、湖边水库、九龙江北溪引水工程等一批水源或流域治理项目完成或加快推进，莲花水库、长泰枋洋水库的前期工作进展顺利。四是加强规划基础工作。建立健全科学规划的决策机制、实施机制和监督管理机制，注重提高规划透明度，注重社会参与。加强违规违章建筑的查处，维护规划严肃性。开展第二次全国土地调查工作，新一轮土地利用总体规划修编大纲通过国土资源部审查。

　　（五）推进改革开放，发展空间更加拓展

　　坚持以改革增活力、以开放拓空间，集聚市内外和国内外资源促进发展、服务发展。一是推进新一轮政府机构改革、事业单位清理改制、中小学教师工资绩效改革；加强国有资产资源整合，完善考核体制；深化政府投资项目后评价工作和公共资源配置市场化改革；完善土地出让“招拍挂”、建设工程招投标、药品和医疗器械招投标、政府采购等管理机制改革。二是打造政策平台。火炬（翔安）B型保税物流中心投入运营，象屿保税区、保税物流园区和东渡港区完成整合并叠加相关政策功能。海沧保税港区面积9.5092平方公里，是国务院批准设立的面积较大的保税港区，是目前我国开放程度最高、功能最齐全、政策最优惠、通关最便捷的海关特殊监管区域，一期工程已建成并通过国家验收。我市还被列为中科院在全国重点布局的技术创新与成果产业化基地、国家计生委全国人口计生综合改革示范城市、科技部首批国家“十城万盏”半导体照明示范工程试点城市。三是加强利用外资，扩大国际交往。实际利用外资22亿美元（按历史可比口径），新引进3个世界500强企业。接待外国代表团160批次。新增2个国际友城。授予17位华侨华人、台港澳人士及外国友人为“荣誉市民”。金龙客车援助塞内加尔项目建成投产，领事馆区一期和福建（厦门）－新加坡友好医疗服务中心项目开工。成功举办第13届中国国际投资贸易洽谈会、国际马拉松赛、国际海洋周、国际动漫节、国际龙舟赛、国际友城市长论坛。厦门爱乐乐团赴美加演出。四是加强对内开放和区域协作。央企和大型民企招商有新突破，合同利用内资470亿元。发挥“海西”品牌效应，推行“无水港”模式，中西部货源腹地进一步拓展。闽西南五市、闽粤赣十三市、与龙岩漳州山海协作等各种区域协作得到加强。

　　（六）加大统筹力度，社会建设更加和谐

　　以改善民生为重点，积极推进岛内外一体化、社会建设和各项社会事业协调发展。被评为“全国推进义务教育均衡发展工作先进地区”，荣获“东亚海岸带可持续发展地方政府杰出成就奖”，在全国重要城市公共文明指数测评中获得第二名。

　　岛外农村发展加快。以推进城镇化、农业产业化为主线，加快岛外开发和农村发展。农副产品与食品加工产业集群实现销售收入195亿元，增长5%，新增3个国家级农业标准化示范区、2家福建省农业企业品牌金奖。同安五峰等乡村旅游试点启动。培育农民专业合作社116家，带动农户4.6万户，“一村一品”注册商标57个。农村富余劳动力转移就业2.4万人。义务教育和公共卫生体系建设加强。实施20个旧村改造、12个老区山区村建设、13个山区村饮水工程，新建改建通行政村和自然村公路143公里，通自然村道路硬化率达75%，公交车通村率达94%，有线电视入户率达95%。新建2个镇级文化中心、20个农村文化室和97个“农家书屋”。基本养老保险新增参保7980人，农村居民参加基本医疗保险62.4万人。

　　社会事业投入加大。全市财政资金投入59.8亿元，增长13.5%。启动中小学校舍抗震安全排查、加固改造工作。完成中小学校新（扩）建14所，新增学位1.6万多个，进城务工人员子女义务教育进入公办校就学比例达67%。免除城乡义务教育各项收费1.17亿元。市妇幼保健院扩建、中山医院内科病房楼、第一医院急诊综合楼等项目建设顺利，完成24个社区卫生服务中心和28个农村卫生所标准化建设，市民健康信息系统在全国率先实现健康信息区域共享，荣获地方政府创新奖。出生人口政策符合率96.9%，低生育水平保持稳定。建成小白鹭艺术中心、同安文体中心。闽南戏曲艺术剧院等项目前期工作加快。南音入选联合国教科文组织“人类非物质文化遗产代表作名录”。成功举办中国戏剧节、全国青少年钢琴比赛、2010年中国新年音乐会、市运会、群众文化艺术节等文体活动。取消公交空调费。开展社区居家养老试点。建设残疾人庇护工场和爱心护理院，改扩建社会福利中心。建成全市缴费信息查询平台。较好完成为民办实事项目。

　　社会保障水平提高。推动出台了我国第一部社会保障性住房管理地方性法规。在2008年提供951套的基础上再向社会提供保障性住房12138套。为全体市民办理自然灾害公众责任险，将全市大学生纳入城乡居民基本医疗保险，完善建筑矿山企业农民工工伤保险。设立道路交通事故社会救助基金。继续提高全市企业退休人员养老金。对就业困难群体实行就业援助和社保补贴，开展厦门本地生源毕业生首次就业（见习）推荐服务。

　　平安建设成效明显。加强社会治安综合治理，信访工作、基层司法调解和多元化纠纷调解工作加强，建立了医疗纠纷第三方调处机制，群众的安全感和对社会治安的满意率提高，被评为全国社会治安综合治理优秀城市。全面落实安全生产责任制，加强专项整治，完善应急管理，防抗台风工作积极有效，岛内降压供水组织严密实施平稳，防控甲型H1N1流感等重大疫情取得阶段性成效。

　　生态建设积极推进。固定资产项目节能评估审查、大型公共建筑和机关办公建筑能耗统计监测、清洁生产、绿色照明等工作有序推进。荣获全国节水型城市。创建国际卫生空港、卫生海港，新增城市绿地700公顷、生态风景林建设1.2万亩，实施成功大道、环岛干道、仙岳路等主干道景观综合整治，完成58万平方米沙滩修复整治。强化机动车尾气、扬尘、工业废气等污染控制，空气质量优良率上升近2个百分点。

　　援建工作进展顺利。支援四川彭州四个镇灾后恢复重建项目30个，总投资8.31亿，已完成25个。对口支援新疆、西藏、重庆、宁夏工作取得较好成效。

　　此外，气象、防震、方志、档案、信息安全等工作得到加强。双拥共建、民兵预备役、人防、海防打私、民族宗教、妇女儿童、老龄、残疾人、红十字会等工作取得新成绩。

　　（七）加强民主法制建设，依法行政更加高效

　　深入开展学习实践科学发展观活动，口岸服务、金融服务、政府部门服务进一步优化，火炬高新区位列全国高新区投资环境竞争力第四名，全市依法行政考评连续三年全省第一。全年提请市人大常委会审议法规草案6项，制定和修订政府规章5项。自觉接受人大及其常委会监督，依法执行人大决定决议，向人大及其常委会报告工作7次31项，办理人大代表议案3件、建议228件，配合人大常委会开展执法检查、人大代表视察及专项调研40多场次。重视和支持人民政协履行政治协商、民主监督、参政议政职能，定期开展政情通报，重大事项注意征求社会意见，办理政协提案468件，其中主席会议确定的重点提案7件。加强科学决策和政务公开。颁布实施《厦门市规范行政处罚自由裁量权规定》，办理行政复议210件。完成了第二次全国经济普查，市政府网站名列省会城市和计划单列市第四名，政府绩效管理、效能建设、审计监督、专项监察、纠风专项治理等工作取得新成效。

　　各位代表！

　　过去一年，我市在克服困难中前行，在抓住机遇中提升，在谋划未来中夯实基础，各方面工作取得了积极成效。这些成绩来之不易，这是党中央、国务院，省委、省政府和市委的正确领导，全市人民齐心协力、艰苦奋斗和各方面大力支持的结果。在此，我谨代表市人民政府，向全市人民，向给予政府工作有力支持和有效监督的人大代表和政协委员、各民主党派、工商联、各人民团体、无党派人士、离退休老同志和社会各界人士，向为厦门发展做出积极贡献的中央和省驻厦单位、驻厦部队、武警官兵和公安干警、投资者和外来建设者，向所有关心支持厦门发展的港澳同胞、台湾同胞、海外侨胞、国际友人，表示衷心的感谢和崇高的敬意！

　　同时，我们也清醒地看到，面对国际金融危机的影响和后危机时代的国际竞争，我市的发展面临着一些亟待正视和需要迫切解决的问题。一是产业发展、结构优化、发展方式转变的问题。去年全市经济增长低于年初预期，有金融危机的影响，也有经济发展产业支撑力不足的问题。随着基础设施的完善和城市空间的拓展，大抓产业项目，加大产业投资比重，尤其是加强先进制造业和现代服务业投资，培育新的经济增长点、财税点、就业点，更有条件也更为紧迫。二是建设创新型城市，培育永续竞争力的问题。有地域、资源限制的因素，也有未能充分有效利用现有资源的问题。随着建设创新型城市，大力提高自主创新能力，培育和壮大有特色的产业显得十分必要。三是经济特区进一步深化体制机制改革的问题。有综合配套的外部条件，也有思想不够解放、政府服务不到位的问题。因此要继续探索、不断创新推动科学发展的体制、机制和软、硬件环境。四是加大力度推进岛外城市化的问题。在推进岛内外一体化过程中已经有了一定的基础、条件，也有认识需进一步统一、措施需进一步加强的问题。在把“三农”工作的重点放在推进岛外工业化、城镇化和农业现代化的同时，要把维护农民合法权益、增加农民收入放在更加突出的位置。五是构建和谐社会的问题。有与经济发展相适应相同步的原因，也有认识和工作不到位的问题。随着经济加快发展，我们应十分重视构建与之相适应、人民满意的社会事业和社会保障体系。上述这些问题，我们将高度重视，并在工作中努力解决。

　　二、2010年工作意见

　　今年是实施“十一五”规划的最后一年，也是为“十二五”规划的实施奠定坚实基础的一年。这一年，巩固应对国际金融危机成果的任务还十分艰巨。一方面，国际国内经济形势逐步好转，国家继续实施积极的财政政策和适度宽松的货币政策，中央支持海西建设和两岸交流合作更趋紧密，发展环境有望好于去年。另一方面，发展中不确定、不可预料的因素和“两难”问题增多，形势仍然比较严峻。我们要始终保持清醒头脑，防止盲目乐观，深刻认识形势的复杂性和做好今年工作的艰巨性，坚定信心，科学谋划，把壮大实力与优化结构、振兴二产与提升三产、新城建设与产业发展、当前发展与长远后劲、经济发展与民生稳定紧密结合，努力推进经济社会平稳较快发展。

　　政府工作的总体要求是：全面贯彻党的十七大和十七届三中、四中全会精神，以邓小平理论和“三个代表”重要思想为指导，深入贯彻落实科学发展观，围绕建设现代化国际性港口风景旅游城市和海峡西岸重要中心城市的目标，按照中央“五个更加注重”的要求和市委工作部署，加快产业发展，建设创新城市，拓展城市空间，服务对台大局，构建和谐社会，在海西建设科学发展之区、改革开放之区、文明祥和之区、生态优美之区中发挥龙头示范作用。

　　主要预期目标为：地区生产总值增长11%；规模以上工业总产值增长11.4%；全社会固定资产投资增长10%；外贸出口增长8%；实际利用外资22亿美元（按历史可比口径）；地方级财政收入增长11%；社会消费品零售总额增长14%；城镇居民人均可支配收入和农民人均纯收入均增长9%；居民消费价格指数103.5%；城镇登记失业率控制在4.5%以内；人口自然增长率控制在9.5‰以内。单位生产总值能耗降低2.52%，二氧化硫排放量下降1.1%，化学需氧量减少0.43%。

　　实现上述目标，我们必须扎实做好以下工作：

　　（一）加快重点产业发展，促进经济发展方式转变

　　把有特色有前景的产业发展作为经济结构调整和经济发展方式转变的抓手，落实到项目上，落实到投资、消费、出口的协同拉动上。

　　继续做强先进制造业。以培育13条先进制造业产业链（群）和培育10个百亿以上产值大企业集团为重点。一是着力抓好现有骨干企业。通过建立联系服务机制，促进企业稳步发展。二是着力抓好工业投资。确保全年完成投资220亿元。乐捷科技、景智光电、玉柴发动机、友达光电四期、厦船三期、建颖科技、百路达卫浴、宸鸿科技园等一批项目要按计划推进，加快投产。新上的项目注重单位面积投资强度、产值、税收以及项目的科技含量，具有产业链高端优势。争取液晶面板等一批在谈重大项目尽快落地实施。三是着力抓好重大项目招商。有针对性开展与跨国公司、央企、台湾行业十强企业、大型民营企业、大型投资公司的对接，通过以商引商，策划生成一批大项目。四是着力抓好基础建设。进一步完善火炬高新区、机械工业集中区、同安工业集中区、环东海域产业园的配套设施，加强视听通讯、钨材料、半导体照明、电力电器等国家特色产业基地建设，广泛支持企业开展技术改造、技术创新，加强高新技术企业认定服务工作。

　　加快做大现代服务业。着力打造有较强影响力和辐射力的区域性现代服务业中心。继续突出10个方面重点，培育一批各行业的龙头企业，鼓励以厦门为基地向外拓展业务。一是加强规划，积极扶持。继续抓好观音山、五缘湾、杏林湾等营运中心，现代物流园区、前场铁路大型货场，软件园、会展三期，思明龙山、湖里老工业区、滨北旧厂房改造等文化创意园区项目建设。根据各行业的特点和发展规律，制定出台有针对性的扶持政策。二是加强推介招商，抓好项目落地。争取设立期货交易中心、期货交割仓库、国家开发银行厦门分行。争取火炬高新区进入“新三板”扩容试点。继续引进一批知名物流企业。推动游艇、帆船、邮轮、高端医疗、温泉生态休闲业的发展。招办新的有规模的展会。争取中国移动通信集团在厦设立手机动漫基地。推进特易购购物中心、电子元器件交易中心、圣果院商业中心等项目实施。三是扩大对外影响力，拓展市外市场。积极开展城市营销。支持在厦金融机构异地设立分支机构。加大力度发展内地“无水港”和海铁联运。加强区域旅游合作。办好第二届世界投资论坛、“投洽会”、“石材展”、“动漫节”等品牌展会。

　　重视发展战略性新兴产业。主动对接国家战略，制定针对性规划和实施方案，培育具备一定产业基础、适合我市发展的战略性新兴产业。新能源领域，重点发展太阳能光伏、生物质能等产业；新材料领域，做大做强特种金属材料、新型建筑材料等产业，加快光电子材料、高分子功能材料的产业化，壮大光电、环保产业基地；生物医药领域，加大戊肝疫苗和抗癌新药等项目研发和建设，鼓励大型医药企业来厦投资设厂，支持申报国家重大新药创制专项；信息网络领域，积极发展“核高基”等产业，促进物联网与互联网的结合，推进传感网的广泛应用；海洋领域，加快发展海洋生物制药、海洋能源资源开发、海洋监测与信息服务等产业，提升厦门国际海洋周效应，建设海洋经济强市。

　　充分发挥投资消费出口带动产业发展的基础作用。一是优化投资结构。在保持投资较快增长的基础上，引导资金重点投向产业发展、重大片区、基础设施、民生保障、生态环境建设等领域。全年安排重点建设项目179个，年度投资363亿元。加强重点项目建设的组织领导，落实重点建设责任制，完善征地拆迁政策。二是努力扩大消费需求。继续打造区域性消费中心，建设度假休闲、商务会展城市、海峡两岸游重要口岸城市。落实国家扩大消费的优惠政策，鼓励汽车消费，继续做好“家电下乡”工作，拓展通信产品、家政服务、体育健身、老年服务、休闲旅游等消费，稳定房地产市场。发展物流配送、连锁经营、电子商务等现代流通方式，促进扩大消费。改善消费环境，发展消费信贷，保护消费者权益。三是提升外贸竞争力。保持外贸进出口总量占全省的份额和比重。加大力度扶持客车、钨制品、游艇等优势产品出口。大力发展流通型出口企业，提高出口产品研发设计水平，争取列入国家设计中心试点城市。支持和引导出口企业打造国际化的自有品牌，加强研发队伍建设，提高产品设计、开发创新能力。帮助企业应对国际市场技术壁垒，加强出口产品的检测认证，支持重点企业建立检测实验室，支持重点行业建立公共技术服务平台和信息服务系统。继续优化通关环境，创新外贸融资机制，扩大信保扶持政策覆盖面，支持企业参加“广交会”等展会，争取我市列入第二批跨境贸易人民币结算试点城市。引导企业调整市场结构，开拓新兴市场，降低集中度。

　　（二）加快建设创新型城市，提高发展的永续竞争力

　　始终把科技进步摆在优先发展的地位，将经济发展进一步转移到依靠科技进步和自主创新的轨道上来，将城市发展融入到经济、社会建设的各个环节。以同时被列入国家发改委和国家科技部“创新型试点城市”为契机，认真落实各项工作，推进“创新城市”建设，不断提升城市综合竞争力和发展的生命力。

　　培育自主创新能力。着力加强区域创新体系建设。一是继续加强重大科技平台建设。在建好原有20个重大科技创新公共平台基础上，重点加快厦门产业技术研究院建设，使之成为国内外高水平科技成果的转化中试基地、新设研发机构和科技企业以及科技中介机构的孵化器，成为市属公共科技服务平台的重要载体、行业关键技术科技攻关的组织协调者和实施场所。二是继续加强以企业为重点的创新主体的培育。大力推动国家、省、市三级创新型企业的认定；加强工程技术研究中心、企业技术中心、重点实验室、博士后工作站、企业孵化器建设；积极组建产业技术创新联盟。三是大力构筑产学研合作体系。深化面向全国重点理工大学的“市校合作”工程和面向中科院及其研究所的“院地合作”工程，积极争取全国理工高校、中科院及其研究院所、跨国公司、大企业集团来我市设立分支机构或研发中心。发挥科技部“对台科技合作与交流基地”的作用，建设对台科技交流合作实验区，对接台湾光电、软件、IC设计、生物与新医药等高新技术产业，积极争取台湾科研机构来厦设立分支机构，共同组建创新团队。四是建立有力的人才保障体系。加快人才创业港建设，实施科技创新创业领军人才引进计划，加强科技人员创新能力继续教育。在资金补助、团队建设、职称评定、工作场所、贷款贴息以及落户、住房、医疗、子女入学、配偶安置等方面为人才创业提供更为便利的条件。五是为发展战略性新兴产业服务。规划建设厦门科技园区，加强信息工业、工业设计、节能产业、环保产业、新材料等战略性新兴产业的研究。

　　积极推行“低碳”发展模式。明确建设“低碳城市”的战略定位。在经济发展上，坚持可持续发展，通过加强技术创新、产业转型、新能源开发、布局低碳化循环化，实现低能耗、低污染、低排放为基础的经济发展。在城市发展上，积极构建绿色交通体系、绿色市政体系，发展绿色建筑，倡导绿色消费。积极开展低碳经济研究，制定相关的统计、评价体系，加强低碳技术开发与应用，逐步建立低碳发展的法规保障体系、政策支撑体系、技术创新体系和激励约束机制。

　　大力发展文创经济。把发展文化创意产业作为提升“软实力”的重中之重，加大政策扶持力度。与实施品牌战略相结合，着力培育一批文化创意产品品牌、企业品牌和行业品牌，培育一批具有全国乃至国际影响力的行业领军品牌、文化创意产业集聚区，着力打造“创意之都”。实施知识产权战略，提升知识产权创造能力，加强知识产权保护工作。鼓励创新、扶持创业，努力在全社会营造崇尚创新、自主创业的良好氛围。

　　（三）加大力度推进岛外开发，提升城市辐射带动力

　　立足城市定位，把做大城市规模，完善城市功能，提升辐射带动能力的重点转移到岛外，统筹产业发展、基础设施、市政建设和生活配套，突出城市特色，打造一批建筑精品和城市亮点。

　　加快启动新城建设。集美区重点依托厦门新站、园博苑和文教区，建设富有文教、科研和交通枢纽特色的新城区；海沧区重点依托港口和产业基础，加快建设大港口，培育大产业、大物流；同安区重点依托海湾、生态及空间优势，把环东海域打造为城市的新增长极；翔安区重点依托海底隧道贯通后形成的新优势，建设与厦门本岛媲美的现代新城。岛内方面，东部片区与旧村搬迁改造相结合，实现会展片区、观音山、软件园、湖边水库、五缘湾的连片发展，打造本岛东部一流商务区；湖里老工业区与发展现代服务业相结合，加快整体改造和转型。同时，继续抓好岛内工业企业搬迁，稳步推进旧城改造，注意保持城市风貌和特色，降低开发强度和建设密度，高标准建设城市综合体。

　　继续加强基础建设。交通基础方面，继续推进龙厦铁路、厦深铁路、厦成高速、厦漳跨海大桥、沈海高速扩建等项目，加快高崎机场三期扩建。深化第二东西通道、轨道交通、新机场等项目前期工作。整合港区资源，推进海沧、招银港区等一批泊位和浏五店航道一期等工程建设。结合岛外新城建设，统筹规划城市交通网络，加快建设厦门新站综合交通枢纽等项目，完善BRT快速公交网络和枢纽站点建设。建成环岛干道，实施厦门火车站改扩建、枋湖客运中心等项目。市政方面，实现东部垃圾焚烧发电厂点火试运行，开工建设西部垃圾焚烧发电厂，续建集美固废中转站，建设城市生活垃圾分类处理厂。加快推进北溪引水左干渠改造二期工程和莲花水库建设，注重水源地涵养林建设与保护，继续做好长泰枋洋水利枢纽工程前期工作。

　　加快推进农村城镇化。通过新城建设、重大片区开发、工业园区建设、重大产业项目等带动和加快岛外农村城镇化步伐。加强规划和空间布局，完善区域交通、市政工程、水资源配置和水利设施、环境保护与防灾减灾等各类专项规划。加强农民转岗就业的组织和服务，使农民收入在城镇化过程中有较快增加。继续推进旧村改造，加强老区山区村建设，实施农村自来水改造、有线电视进村入户和电影放映工程，加快农村道路建设向自然村延伸，进一步提高农村客运覆盖率。推进水库除险加固等水利设施建设，实施同安东溪流域下游河道、翔安九溪流域整治，完成过芸溪、深青溪、瑶山溪治理。

　　（四）加强对台交流合作，充分发挥前沿平台作用

　　认真落实中央对台工作的统一部署，充分发挥“五缘”优势，深化两岸交流合作，拓展作为空间，建设前沿平台，服务大局、服务两岸。

　　继续先行先试。推进台商投资区扩区和功能整合，争取成为实施两岸经济合作框架协议（ECFA）试点城市和两岸金融合作试验区，在两岸金融机构互设、金融从业证照互认和货币双向兑换、跨境贸易人民币结算与两岸货币清算等方面先行先试。推动厦门居民赴金、马、澎旅游自由行，争取实行在厦暂住人员在厦办证赴金门旅游。大力开展海上邮轮业务，争取开辟以厦门为母港的台湾海峡邮轮航线。推动厦门机场成为两岸货运包机新增航点。做好厦门邮件封发局运营工作，推动在厦设立对台邮包交换中心。加快推进厦金直接通信工程建设，积极开展向金门供电、厦金通道等项目前期工作。密切厦台、厦金海上通航和救援合作。

　　着力抓好对接项目的实施。围绕“十对接”，打造对台产业对接集中区、对台服务外包合作示范城市、对台科技合作交流基地。继续支持办好现有台资企业，大力引进台湾企业来厦设立地区总部、配套基地、采购中心、物流中心、营运中心和研发中心。加强与台湾科技园区的对接合作。扩大厦金航线功能，培育厦澎航线，吸引更多两岸同胞来厦旅游或经厦往来两岸旅游。加快大嶝对台小额商品交易市场扩建，推进长庚医院二期建设，推动金门酒厂总部大厦、五缘湾台湾形象馆等项目落户。

　　积极支持两岸的广泛交流。精心筹备办好第二届海峡论坛，丰富活动内容，凸显品牌效应。争取国家部委、两岸协商机构、台湾行业协会来厦设立办事机构，建设两岸事务重要协商地。深化厦台之间教育、文化、卫生、体育、民俗、宗教等方面的交流，加快建设海峡中医药合作发展中心和海峡两岸中医药博物馆，推动厦门媒体赴台设点。推动涉台法规的修订完善，依法保护台胞正当权益。继续办好台交会、文博会、图书交易会等两岸各类展会活动。

　　（五）深化改革扩大开放，增强发展的动力和活力

　　进一步发挥经济特区优势，把内生型发展与开放型发展、深化改革与扩大开放结合起来，在更大范围、更广领域、更高层次上增强体制机制活力，集聚发展资源。

　　继续争取重大政策。充分发挥国家赋予的经济特区作为体制机制创新试验区的优势，在原有工作基础上，继续争取经济特区范围延伸到全市、实施综合配套改革、台商投资区扩大范围、设立两岸金融合作试验区等重大政策，争取成为国家对台服务外包合作示范城市。

　　研究制定建设创新型城市的政策体系。围绕创新型城市的目标、任务，把政府的组织引导与市场机制结合起来，制定相关的组织保障机制、政策保障机制、资金保障机制、人才保障机制。特别是加快发展创业风险投资，积极发展资本市场，推动科技与金融合作。

　　探索城镇化发展的制度创新。着力于服务加快岛外城市化进程的目标，加快农村“三资”清理确权、有序推进“村改居”社区集体资产股份量化。推进农村社区股份合作社建设，完善“金包银”管理运行机制。探索建立城乡建设用地增减挂钩制度，规范农村土地承包经营权流转。推动户籍制度改革，逐步实现岛内外的户籍制度一体化。

　　发挥市场竞争机制的导向作用。着眼于做大做强现有企业，鼓励有条件的国有企业集团和各类企业发挥品牌、管理、技术、队伍、基础等各种优势，积极参与市外、省外竞争和国际竞争。继续加快非公有制经济发展，落实鼓励民间资本进入基础设施建设、公用事业、金融服务、社会事业等领域的相关政策，保护民间投资合法权益，支持各行业民营龙头企业、骨干企业做大做强。打破所有制界限，政府支持发展的政策以产业、行业、区域为标准。大力发展各行各业的同业协会组织，加强行业自律和服务，维护公平竞争，促进共同发展。

　　加强国际经贸交流和区域协作。充分发挥海沧保税港区等政策性平台的作用，集聚企业、项目和各种高品质的发展资源。面向欧美日等发达国家和台湾地区，加强跨国公司、500强企业、知名研发机构的招商。密切与港澳合作，创新经贸协作形式和载体。借助中国－东盟自贸区平台，加强与东南亚国家的投资合作。深化与国际友城、友好港口的交流合作。办好第八届世界同安联谊大会。继续加强与央企、大型民企、高等院校、科研院所的合作，与长三角、珠三角地区的经济协作和技术协作。密切海西城市群、闽粤赣十三市、闽西南五市的全方位合作，加强与全国各地闽商商会、海外华人华侨商会的联系。继续做好西藏、新疆、宁夏、重庆等对口支援工作，全面完成四川彭州四镇灾后援建任务。

　　同时，认真落实国家和省里统一部署的有关机构改革、投融资体制改革、财税体制改革、审批制度改革、事业单位人事制度改革、医药卫生体制改革、文化体制改革、价格改革以及政府管理、公共服务、内部建设等方面的体制机制改革，立足实际，先行先试，积极探索。

　　（六）努力构建和谐厦门，持续创建美好家园

　　加强强农惠农政策的落实。认真贯彻落实中央、省、市支农扶农各项政策措施，加快建立城乡基础设施共同发展机制、城乡公共服务均等供给制度、城乡衔接的社会保障体系。加大力度培育、壮大农副产品与食品加工产业集群，支持发展“一村一品”特色农业、休闲农业和生态农业。加强农业防灾减灾体系建设，完善防汛抗旱、森林防火、动植物防疫等预警预报机制，增强气象防灾减灾和应对气候变化能力。

　　加强就业服务和社会保障。完善就业服务体系，加强困难群体就业援助，适度提高最低工资标准。落实被征地农民和上岸渔民享受城镇职工就业再就业政策，加大订单式培训和公益性岗位开发，鼓励自主创业，力争完成农村劳动力转移15万人。推进“全民社保城市”建设，实施新型城乡居民基本养老保险办法，认真做好全国城镇企业职工养老保险跨地区转移接续工作，完善外来人员基本养老保险办法。继续提高参保职工基本养老保险金，适当提高医疗、工伤、生育保险待遇水平。推进建筑施工企业和商贸、餐饮、旅馆等服务行业以及个体工商户参加工伤保险。继续完善居民医疗保险制度，逐步将符合条件的村卫生室以及高校内设医疗机构纳入医保定点服务范围，方便居民就医看病。继续做好社会保障性住房建设项目的实施，着力解决中低收入群众住房困难。整顿和规范房地产市场秩序，增加普通商品住房供应，支持居民自住和改善型住房消费，遏制投机性购房。

　　加强社会事业发展。教育方面，继续推进义务教育均衡发展，全面推进素质教育，加强教师队伍建设，完善招生制度。抓好义务教育学校标准化建设、校舍安全工程建设和集美、翔安文教区建设。继续共建厦大、集大，确保厦门理工学院通过教育部本科教学评估。重视发展学前教育、特殊教育、双语教育。卫生方面，继续加强公共卫生体系和农村卫生工作，推进基层医疗卫生服务机构标准化建设，加强乡村医生、全科医生培养力度。大力培育“名院、名科、名医”，扶持社会医疗机构健康发展。实施仙岳医院扩建、翔安医院、五缘医院建设等项目。落实人口计生综合改革示范市建设各项工作，稳定低生育水平。文体方面，抓好文体惠民工程的实施，健全文体服务网络，兴建和完善一批重点文体设施。加快闽南文化生态保护实验区建设，推进鼓浪屿申报世界文化遗产工作。完成青少年宫项目建设，开工建设市运动训练中心等项目。广泛开展全民健身活动和校园阳光体育活动，办好国际马拉松赛、全国青少年大提琴比赛、中国国际钢琴比赛、鼓浪屿钢琴节。继续加强其他公共事业发展。优化公交线网布局，加强出租车市场管理。完善社会救助体系，支持发展各类慈善公益事业，做好扶老、救孤、助残、济困工作。努力解决来厦务工人员的社会保障、子女就学等问题。认真组织实施为民办实事项目。

　　加强生态城市建设。积极推进“健康城市”、“国家环境保护模范城市”、“国家森林城市”建设。落实节能减排年度目标，推进国家“十城千辆”节能与新能源汽车应用示范工程和“金太阳示范工程”，加强公共机构节能工作，推广节能示范工程项目，更新使用天然气公交车；完善监控措施，确保重点工业企业污染物达标排放；加大主要流域污染整治力度，有效控制畜禽养殖等污染。完善数字城管系统，加快机动车简易工况排气检测中心等项目建设，加强环境卫生、市容市貌、交通秩序以及汽车尾气、工业废气、扬尘污染、油烟噪声扰民治理的日常管理；加强城市绿化、美化、亮化、彩化工作，继续开展城市主干道和重要城市节点景观整治，实施厦门莲花国家森林公园建设等项目，全年新增绿地700公顷、林相改造1万亩。加强资源和环境保护，继续推进海域清淤整治和沿海防护林体系建设，巩固筼筜湖治理成果，开口改造高集、集杏海堤；落实重要水源地生态保护补偿机制；节约集约用地，盘活存量土地，提高土地利用率。

　　加强文明城市创建工作。以社会主义核心价值体系建设为根本，积极推进思想道德和精神文明建设。认真落实《创建文明城市三年规划》。充分发挥社区、居委会和各群团组织、行业协会、社会团体的作用，深入开展社区和谐、村镇新风、行业满意、军民共建等创建活动。加强社区建设，提升社区物业管理水平。加强流动人口的管理服务，共建文明温馨家园。

　　维护社会安定稳定。加强治安防控体系建设，严厉打击刑事犯罪，及时解决突出的治安问题。落实安全生产责任制，强化专项治理，加强大型桥隧和重大危险源安全管理。加强市场监管，确保食品药品安全。加强市场监测，确保重要农副食品和公用行业公共服务价格稳定。加强网络信息工作，认真做好信访和人民调解工作，防止各类不稳定事件发生。加强民族与宗教工作。加强诚信体系建设。加强劳动保障执法监察，健全完善劳动争议调解仲裁机制。整合防空防灾资源，加强应急管理和指挥平台建设，提高应急救援和突发公共事件的社会救助能力。做好甲型H1N1流感等疫情防控工作。

　　三、持续加强自身建设，着力提升服务水平

　　牢记特区使命，抢抓发展机遇。进一步解放思想，先行先试，以更宽的视野、更高的站位，做好今年的各项工作，组织编制好“十二五”规划。以更加奋发有为的精神状态，更加富有创新的举措，更加积极有效的作为，发挥厦门经济特区在体制机制创新方面的试验区作用，在海西建设中的龙头示范作用。

　　转变政府职能，提升服务效能。认真履行社会管理和公共服务职能，努力为各类市场主体创造良好发展环境、提供优质公共服务。建立健全政府联系企业、服务企业的长效机制，继续开展以“五帮”为主要内容的“走进和服务千家企业”活动。加强机关效能建设，完善政府绩效评估指标体系。加强政务公开、政府信息公开和电子政务建设，努力把厦门建设成为政府效能最高、行政成本最低、服务质量最好的城市之一。

　　坚持依法行政，优化法治环境。创新政府立法工作机制，进一步强化政府法制监督，严格行政执法责任制和过错追究制度，规范行政自由裁量权，加强行政复议工作，维护社会公平正义。自觉接受市人大及其常委会的法律监督和工作监督，自觉接受市政协的民主监督，坚持定期向市人大及其常委会报告工作和向市政协通报情况。认真听取各民主党派、工商联、无党派人士和各人民团体的意见。认真做好人大代表议案、建议和政协提案的办理工作。支持工会、共青团、妇联等人民团体依照法律和各自章程开展工作。重视新闻舆论和社会公众监督。

　　转变机关作风，狠抓工作落实。坚持深入群众，贴近群众，依靠群众，服务群众。不搞形象工程，不搞形式主义，减少会议、文件、检查评比，一切工作注重实效。坚持重大事项集体讨论决定，完善社会公示、听证和专家咨询、评估机制。进一步健全政府职责体系，加强条块之间的沟通合作，密切部门之间的协调配合。加强公务员法律、政策、专业知识学习，以及对新领域、新课题的研究探索，增强推动工作、服务民众的能力。

　　继续艰苦奋斗，保持清正廉洁。加强从源头上防范和治理腐败，健全权力运行监控机制。认真落实“一岗双责”，加强审计监督，健全政府重大投资项目公示、重大项目效能监察、重大项目后评估、重点事项提前介入等制度。加强重点领域专项治理。深入开展纠风工作，坚决纠正损害群众利益的不正之风。发扬艰苦奋斗、勤俭办事的精神，狠抓增收节支，厉行节约，切实做到为民、务实、清廉。

　　各位代表！厦门的发展，在挑战中再次迎来了新的机遇，站在历史的新起点，面对全市人民的新期待，我们深感责任重大、使命光荣。让我们更加紧密地团结在以胡锦涛同志为总书记的党中央周围，深入贯彻落实科学发展观，在中共厦门市委的坚强领导下，坚定信心，同心协力，攻坚克难，开拓创新，推动厦门经济社会又好又快发展，为把厦门建设成和谐文明的美好家园而努力奋斗！
